# Epidemiological and comparative genomic analysis of *Bacillus anthracis* isolated from northern Vietnam

**DOI:** 10.1371/journal.pone.0228116

**Published:** 2020-02-21

**Authors:** Thi Thu Ha Hoang, Duc Anh Dang, Thanh Hai Pham, Minh Hoa Luong, Nhu Duong Tran, Tran Hien Nguyen, Thuy Tram Nguyen, Tran Tuan Nguyen, Satoshi Inoue, Shigeru Morikawa, Akiko Okutani

**Affiliations:** 1 National Institute of Hygiene and Epidemiology, Hanoi, Vietnam; 2 Ha Giang Center for Disease Control, Ha Giang, Vietnam; 3 Department of Veterinary Science, National Institute of Infectious Diseases, Tokyo, Japan; Zhejiang University, CHINA

## Abstract

To understand the epidemiological and genetic background of anthrax cases occurring in Vietnam from 2011 to 2015, we surveilled and genetically analyzed *Bacillus anthracis* isolated in the north of the country. Epidemiological surveillance showed that most human cutaneous anthrax cases occurred in association with animal dissection. Whole-genome sequences were obtained from six *B*. *anthracis* strains from human patients with cutaneous anthrax in the endemic area. Comparative genomic analysis showed that the genetic homogeneity among Vietnamese *B*. *anthracis* strains was very high. All Vietnamese *B*. *anthracis* strains belonged to the canSNP lineage of A.Br.011/009, which mostly consists of strains of the trans-Eurasian (TEA) group, including the most closely related strain, Carbosap. To clarify the genetic diversity of Vietnamese strains and strains belonging to A.Br.011/009 and A.Br.008/011 canSNP lineages, we applied a reference genome-based single-nucleotide polymorphism (SNP) and gene-by-gene genomic analysis (whole-genome MLST) strategy. The phylogeny from core genome SNPs revealed that the Vietnamese strains were positioned close to each other; moreover, several SNPs specific to Vietnamese *B*. *anthracis* were identified. Whole-genome MLST analysis revealed the differences in the number of SNPs between Vietnamese strains, which could enable discrimination at the strain level.

## Introduction

Anthrax is a severe zoonotic disease caused by the spore-forming bacterium *Bacillus anthracis*. *B*. *anthracis* can survive in the environment for decades as a dormant and stable spore [1, 2]. Herbivores are susceptible to *B*. *anthracis* infection when grazing on contaminated grass. Naturally occurring human anthrax infections are caused by contact with infected animals or animal products (cutaneous anthrax), ingestion of undercooked infected meat (intestinal anthrax), or lung exposure to spores (inhalational anthrax) [3]. Recently, another form of anthrax infection among heroin users in Europe (injection anthrax) was reported [4].

In Vietnam, anthrax has a wide distribution and remains one of the most serious emerging infectious diseases. It is one of 28 infectious diseases that must be reported in the infectious disease surveillance system of Vietnam’s Ministry of Health (MOH). From the surveillance conducted by Vietnam’s MOH, 20–80 human anthrax cases are reported annually, most of which are from northern mountainous regions of the country. Recently, anthrax cases appear to have been concentrated in the mountainous border regions in the north of the country, including Ha Giang, Dien Bien, Lai Châu, Son La, Cao Bang, and Lao Cai provinces [5]. To understand the epidemiological and genetic background of anthrax cases occurring in Vietnam from 2011 to 2015, we surveilled and genetically analyzed *Bacillus anthracis* isolated in northern Vietnam. Epidemiological surveillance showed that most human cutaneous anthrax cases occurred in association with animal dissection. Whole-genome sequences were obtained from six *B*. *anthracis* strains from human patients with cutaneous anthrax in the endemic area. Comparative genomic analysis showed that the genetic homogeneity among Vietnamese *B*. *anthracis* strains was very high. All Vietnamese *B*. *anthracis* strains belonged to the canSNP lineage [6] of A.Br.011/009, which mostly consists of strains of the TEA group [7], including the most closely related strain, Carbosap. To clarify the genetic diversity of Vietnamese strains and strains belonging to A.Br.011/009 and A.Br.008/011 canSNP lineages, we applied a reference genome-based single-nucleotide polymorphism (SNP) and gene-by-gene genomic analysis (whole-genome MLST) strategy. The phylogeny from the core genome SNPs revealed that the Vietnamese strains were positioned close to each other; moreover, several SNPs specific to Vietnamese *B*. *anthracis* were identified. Whole-genome MLST analysis revealed the differences in the number of SNPs between Vietnamese strains, which could enable discrimination at the strain level. In the period from 1991 to 1995, three fatal cases out of 441 anthrax patients were reported from northern Vietnam, according to the MOH. The number of cases decreased in the period from 1996 to 2000 to 219, with no fatal cases. Among these latter cases, 211 were reported from northern parts of Vietnam and 8 from elsewhere in the country (southern Vietnam, 4 cases; central highlands, 3 cases; and central Vietnam, 1 case), according to a Health Statistical Profile by the MOH. From 2006 to 2011, three lethal cases out of 413 anthrax patients were reported in the northern provinces, with the most cases recorded in Dien Bien province. Human anthrax cases were reported year-round, but more occurred in the rainy season from June to September. Almost all patients were aged over 15 years, with 37.9% and 36.1% of patients aged 30–49 and 15–29 years old, respectively. The ratio of male to female patients was 7:3 [5]. More than 98% of human patients presented with skin lesions compatible with cutaneous anthrax. The numbers of intestinal anthrax and septicemia cases were very small, accounting for two and one of the fatal cases, respectively. Most cutaneous anthrax patients had a history of contact with sick or dead cattle, buffalo, horse, goat, and other animals through slaughtering or eating them. In one case, a cow had died suddenly. The owner had then opened the carcass and dressed the meat, which was then sold within the village for human consumption. Transportation of infected meat to neighboring villages also occurred, resulting in the spread of disease. To date, we have isolated *B*. *anthracis* only from lesions of human patients with cutaneous anthrax, but not from environmental sources. The prolonged *B*. *anthracis* spore phase slows the evolution of this species, resulting in high levels of genetic homogeneity [8]. Recently, single-nucleotide polymorphisms (SNPs) have been identified in *B*. *anthracis* and global and regional patterns of diversity have been described using canSNP typing [9] and core genome SNPs from *B*. *anthracis* whole-genome nucleotide sequences [10, 11]. The genetics of *B*. *anthracis* as determined using whole-genome analysis has been widely reported recently in the form of comparative genomic analysis with isolates from many countries. One of the most common methods used to date has been core genome SNP analysis. It is possible to use comparative whole-genome analysis as an unbiased approach in order to discover informative SNPs and trace the origin and previous spread of anthrax [12]. Whole-genome sequencing and phylogenetic analysis of isolates obtained globally can also be used to generate hypotheses about the epidemiological connections among geographically disparate isolates and global patterns of anthrax dispersal [13]. It is also extremely useful to perform national sampling studies of *B*. *anthracis* collections to reveal the actual origin of a strain and identify it accurately [13, 14]. Comparative genome analysis and epidemiological surveillance have also been suggested to aid administrative actions, such as restrictions on the import of livestock products and animals, and thereby help to reduce anthrax cases [15]. SNP typing and subsequent phylogenetic analysis of *B*. *anthracis* strains isolated from worldwide anthrax cases have resulted in the identification of their genetic background [7, 13, 16, 17]. In the present study, whole-genome sequences of six Vietnamese *B*. *anthracis* strains isolated from human cutaneous anthrax patients were obtained for *in silico* canSNP typing and comparative genome analysis.

## Materials and methods

### Bacterial strains and DNA extraction

Vietnamese *B*. *anthracis* strains used for whole-genome sequence analysis are listed in the Table 1 with epidemiological data. The reference strains of *B*. *anthracis* from other countries used in the present study belonged to A.Br.011/009 and A.Br.008/011 canSNP lineages are listed in S1 Table.

**Table 1:** Profiles of six Vietnamese *B*. *anthracis* strains belonging to A.Br.011/009 canSNP lineages.

Strain name	Isolation site	Isolation year	Isolation source	Accession number	canSNP lineage
TuanDB	Phinh Sang commune, Tuan Giao district, Dien Bien province, Vietnam	2011	cutaneous anthrax	DRR166487	A.Br.011/009
DB	Phinh Sang commune, Tuan Giao district, Dien Bien province, Vietnam	2011	cutaneous anthrax	DRR166482	A.Br.011/009
LaLC	Than Uyen district, Lai Chau province, Vietnam	2011	cutaneous anthrax	DRR166484	A.Br.011/009
QuyetLC	Than Uyen district, Lai Chau province, Vietnam	2011	cutaneous anthrax	DRR166486	A.Br.011/009
LamDB	Phinh Sang commune, Tuan Giao district, Dien Bien province, Vietnam	2013	cutaneous anthrax	DRR166485	A.Br.011/009
HG	Niem Tong commune, Meo Vac District, Ha Giang province, Vietnam	2015	cutaneous anthrax	DRR166483	A.Br.011/009

*B*. *anthracis* strains were isolated from skin lesions of patients with typical symptoms of cutaneous anthrax. Biopsy samples from skin lesions of each patient were directly cultured on 5% sheep blood agar at 37°C overnight and a single colony was isolated as an independent strain. The colonies were identified using biochemical tests and PCR [18].

For DNA extraction, the strains were incubated overnight at 37°C on sheep blood agar/TSMP plates. One loop of the bacterial cells from the agar plates was inoculated into Luria–Bertani broth and grown overnight. Each overnight culture was lysed in lysis buffer (QIAGEN, Hilden, Germany) for 30 min at 37°C. Proteinase K was added at a final concentration of 1 mg/mL and incubated overnight at 56°C. RNase A was added at a final concentration of 20 mg/mL and incubated at 25°C for 2 min. Chromosomal DNA was obtained using the standard phenol–chloroform extraction method and was recovered in the aqueous phase after 20 min of centrifugation at 15,000 × g [19]. DNA was precipitated by the addition of two volumes of ice-cold 95% ethanol and collected by centrifugation for 20 min at 15,000 × g. The DNA was then dissolved in MilliQ water or TE buffer (10 mmol/L Tris and 1.0 mmol/L EDTA, pH 8.0) and filtered through a 0.2-μm filter (Millipore, Billerica, MA). The quantity and quality of DNA were analyzed by NanoDrop (Thermo Fisher Scientific, Waltham, MA).

### Whole-genome sequencing of Vietnamese isolates

The preparation of a genomic library with a 250-bp or 300-bp read length was performed using the NEBNext DNA Library Prep Master Mix kit for Illumina (NEB, Ipswich, MA) with index primers from NEBNext Multiplex Oligos for Illumina (NEB), in accordance with the manufacturer’s protocol. DNA library fragments were separated using Agencourt AMPure XP magnetic particles (Beckman Coulter, Brea, CA). Library quality and concentration were determined using BioAnalyzer (Agilent, Santa Clara, CA) with a High Sensitivity DNA Chip Set (Agilent). The libraries were applied to Illumina MiSeq (Illumina, San Diego, CA) using 250 or 300 paired-end sequencing with the MiSeq Reagent Kit v2 (500 cycles) or v3 (600 cycles) for whole-genome sequencing, respectively.

Quality assessment of the obtained reads was performed using the default parameters of CLC Genomic Workbench ver.9.5.3 (QIAGEN). Quality-filtered sequence data from the isolates were subjected to *in silico* canSNP typing determined based on 13 published canonical SNPs [8], as previously reported [15]. SRA files of *B*. *anthracis* belonging to A.Br.008/011 and A.Br.011/009 canSNP lineages for comparison with Vietnamese isolates were obtained (Table 1) and converted to fasta or fastq files using SRA toolkit with the command fastq-dump. The nucleotide sequence data of the Vietnamese *B*. *anthracis* isolates determined in the present study are available in the DDBJ Sequenced Read Archive (accession numbers DRR166482–DRR166487) and as assembled genomes (accession numbers BLET01000001-BLET01001786(TuanDB), BLEU01000001-BLEU01000153(DB), BLEV01000001-BLEV01000162(HG), BLEW01000001-BLEW01000130(LaLC), BLEX01000001-BLEX01000016(LamDB), BLEY01000001-BLEY01000118(QuyetLC)).

### Comparative genomic analysis

We used the Parsnp analytical tool from the Harvest Suite software for fast multiple alignment of genomic sequences [20]. Assembled contigs with the default parameters using CLC Genomics Workbench version 11.0.1 (QIAGEN) were used as input for Parsnp analysis (parameters -c -x). The detected SNPs were extracted to a VCF file, using HarvestTools (version 1.0) from the same software package. To improve the overall quality of the data, any SNPs less than 10 bp away from other SNPs, as well as positions that carry an unspecified nucleotide (“N”), were removed. The edited file was used as an input file in HarvestTools to compile the fasta file.

Assembled contigs of Vietnamese strains and five strains from the TEA group [7] were applied to BacWGSTdb [21] for determining the phylogenetic relatedness among the user-uploaded multiple genome sequences, following both SNP and whole-genome multi-locus sequence type (wgMLST) approaches.

### Ethical approval

The study protocol was reviewed by the local ethics committee at the National Institute of Hygiene and Environment (NIHE) with approval numbers 01 IRB and 14 IRB and at the National Institute of Infectious Diseases (NIID) with approval number 955.

## Results

### Epidemiological characteristics of Vietnamese anthrax

**Fig 1:**
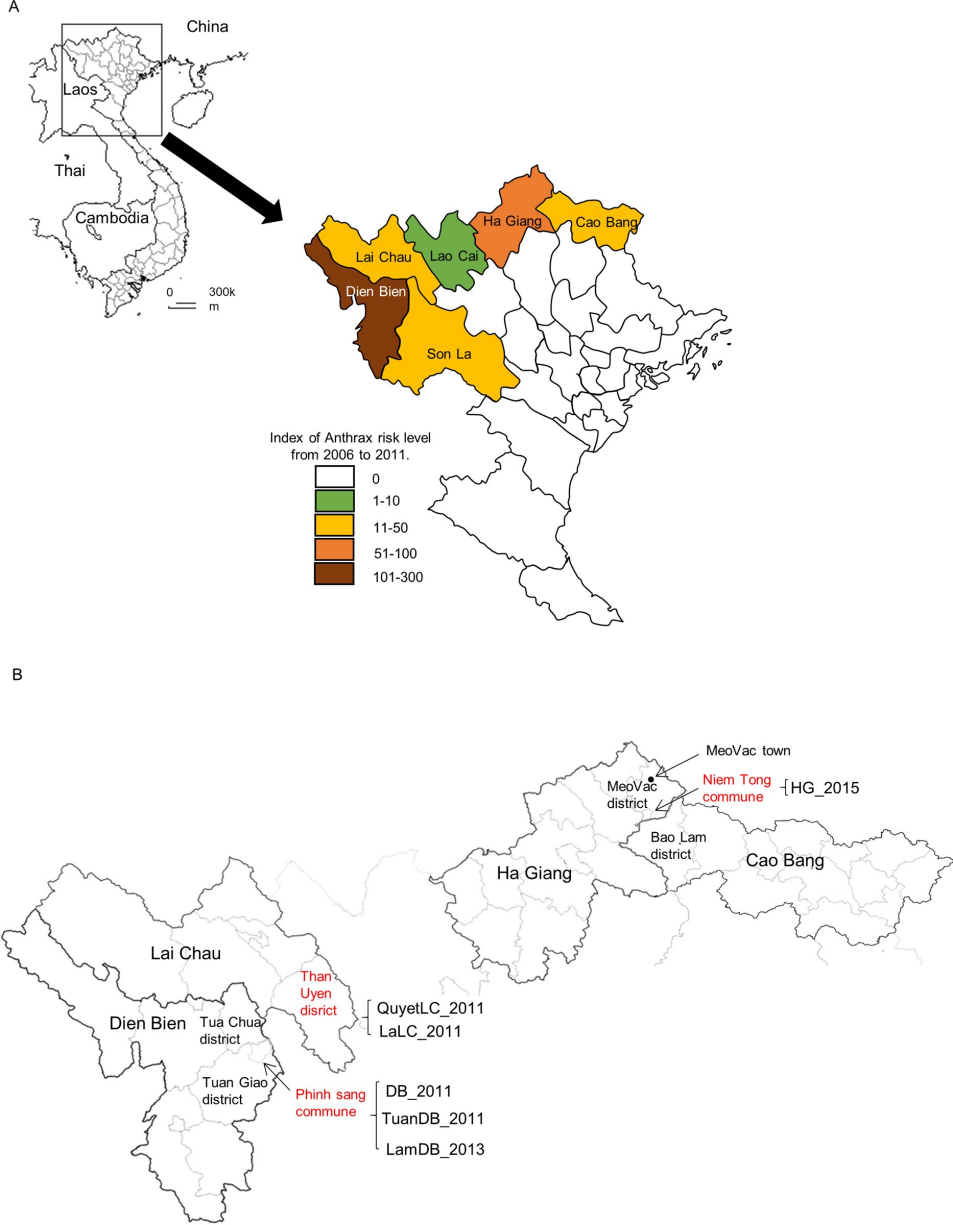
Epidemiological and geographical information about Vietnamese anthrax. (A) Map of Vietnam showing the distribution of human anthrax in northern provinces from 2006 to 2011. The color indicates the human anthrax risk level depending on the number of human anthrax cases occurring from 2006 to 2011 [5]. (B) The anthrax hotspot commune and district are shown. The isolation sites of strains used in the present study are indicated in red, from Dien Bien, Lai Châu, and Ha Giang provinces in northern Vietnam.

**Fig 2:**
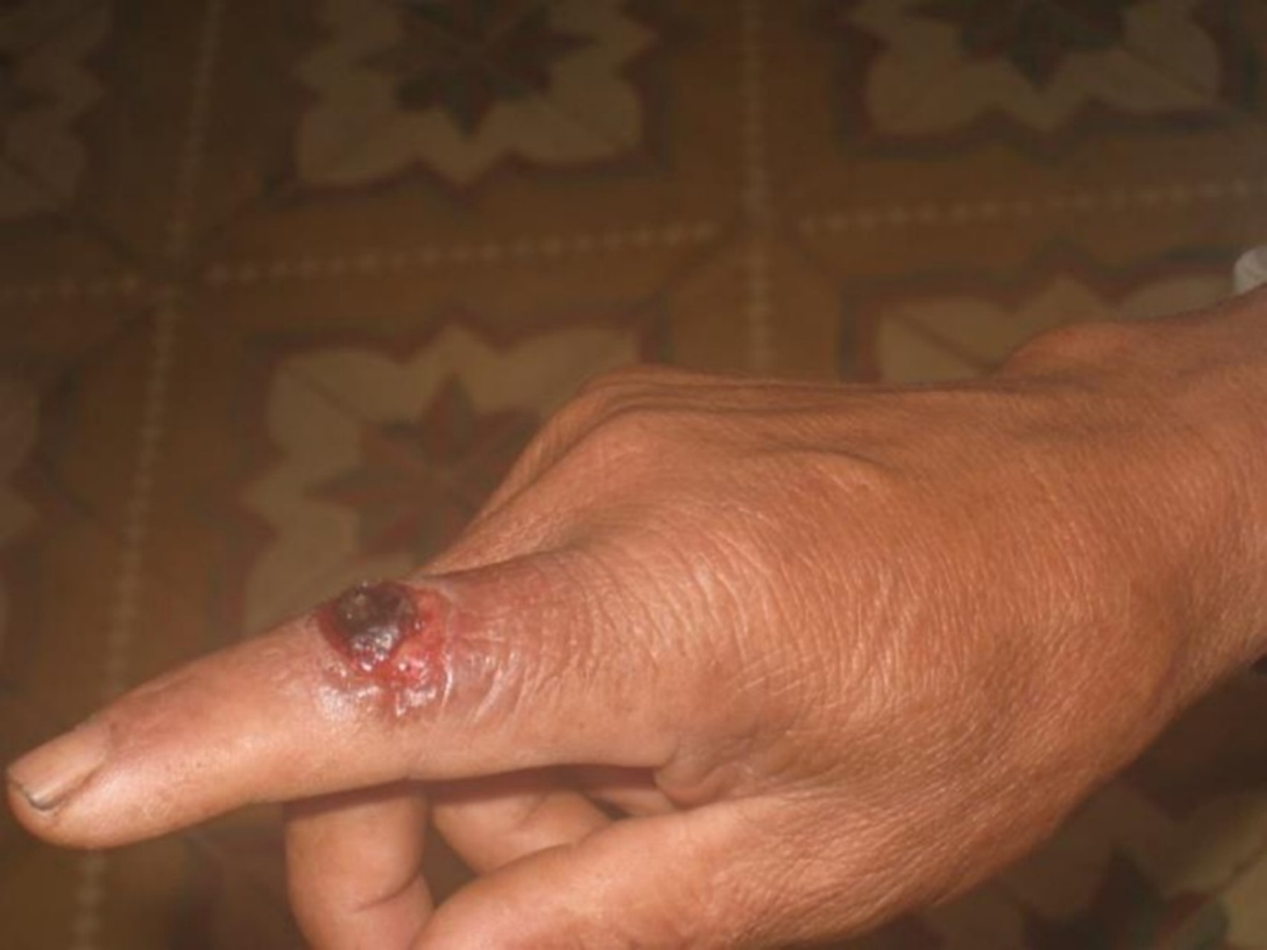
The affected finger of a cutaneous anthrax patient. A typical black eschar on the finger of a cutaneous anthrax patient who had a history of contact with *B*. *anthracis*-infected animals and carcasses.

Recently, we have observed that annual anthrax outbreaks occur in northern Vietnam during the period of heavy rainfall from June to September. This region includes six provinces that are close to the Chinese and Laos borders (Fig 1A). These anthrax outbreaks spread to neighboring villages, where both humans and animals can become infected. The predominant form of human anthrax is cutaneous and includes skin lesions with black eschars on the patients’ necks, fingers (Fig 2), arms, and feet. Diagnosis, by a medical doctor at the Center for Protective Medicine in each province, has usually been based on this typical symptom.

In the study period from 2011 to 2015, 155 suspected anthrax cases were reported based on clinical symptoms. Hotspots of anthrax infection in the communities in the northern provinces included Niem Tong commune, Meo Vac town in Meo Vac district in Ha Giang province, Tuan Giao and Tua Chua districts in Dien Bien province, Than Uyen district in Lai Chau province, and Bao Lam district in Cao Bang province (Fig 1B). Human patients were usually treated with traditional herbal medicines at home and upon admission to hospital. In cases with more severe symptoms, patients were treated with the antibiotic ciprofloxacin. Most patients showed clinical features of cutaneous anthrax. Seven patients had acquired infection through contact with animals, while for the others no clear exposure information was available. A total of 50 eschar and vesicular fluid samples were collected from the patients, to identify and confirm the infectious agent by laboratory testing. Six *B*. *anthracis* strains were isolated from these clinical samples, including three isolates from including three isolates from Dien Bien province, two from Lai Chau province, and one from Ha Giang province. (Fig 1B). All of them were isolated from the cutaneous anthrax patients (Table 1 and Fig 1B).

### Phylogenetic analysis of *B*. *anthracis* using core genome SNPs and wgMLST from whole-genome sequences

**Fig 3:**
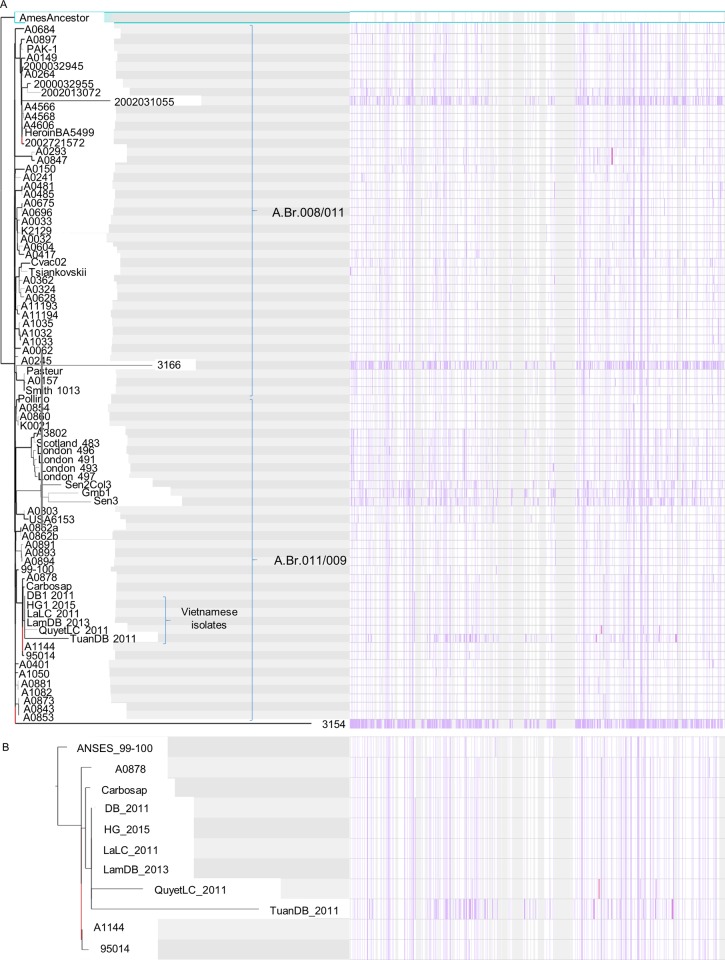
Phylogeny and multiple alignments of *B*. *anthracis* strains belonging to A.Br.008/011 and A.Br.011/009 canSNP lineages. A total of 4,976 core genome chromosomal SNPs (S3 Table) from 82 strains were extracted using Parsnp. (A) The phylogenetic tree and SNP heatmap showing SNP density across the genome of each strain of the A.Br.008/011 and A.Br.011/009 canSNP lineages used in the present study. The Ames Ancestor strain was used as a reference and an outgroup. (B) The phylogenetic tree and SNP heatmap of TEA group strains and Vietnamese strains. Screenshots of the phylogenic tree and SNP heatmap were obtained as output by Gingr.

**Fig 4:**
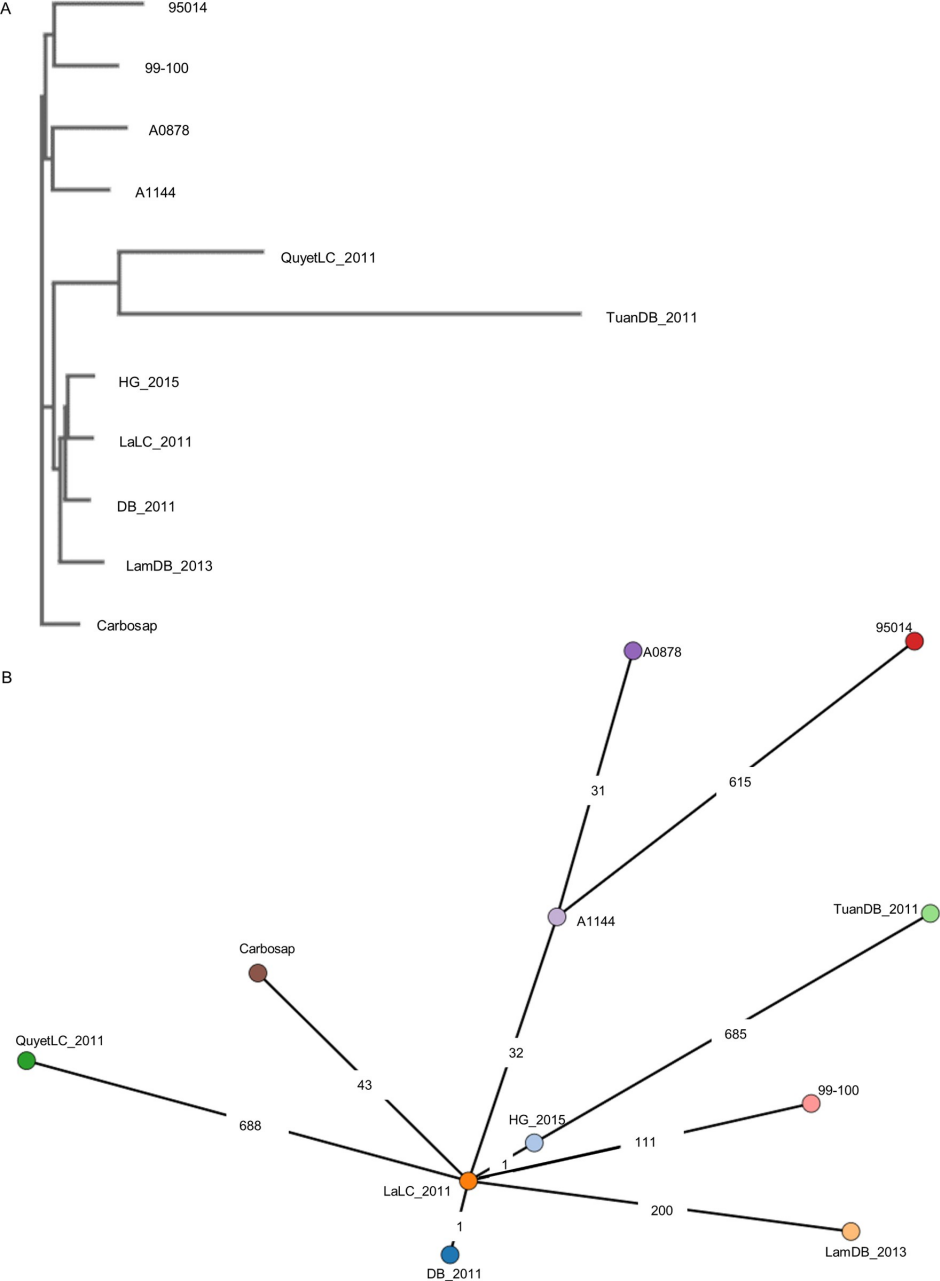
Multiple genome analysis was used to determine the phylogenetic relatedness among the genome sequences of TEA group strains following both SNP and whole-genome MLST approaches using tools in BacWGSTdb. (A) The phylogenetic tree based on the SNP strategy from the assembled contigs of six Vietnamese strains and five genetically closest TEA group strains (Carbosap, A0878, 95014, A1144, and ANSES_99–100) was visualized using the neighbor joining method. (B) Minimum spanning tree based on the whole-genome MLST strategy encompassing not only core genome genes/loci but also accessory genes/loci was visualized with static redraw and log scale modes. The numbers on the branches indicate the number of SNPs between strains.

In the present study, we determined the whole-genome sequences of six Vietnamese *B*. *anthracis* isolates. These six isolates were shown by *in silico* canSNP analysis to belong to the A.Br.011/009 canSNP lineage. Assembled contig files constructed with the CLC Genomic Workbench with the default parameters were used for rapid core genome multiple alignment using Parsnp with 76 strains from other countries belonging to the A.Br.011/009 and A.Br.008/011 canSNP lineages and the Ames Ancestor genome as a reference (Fig 3A). Total coverage among all sequences was 56.4% by Parsnp analysis. The phylogeny using the maximum likelihood method with 4,976 core genome SNPs revealed that an Italian strain, Carbosap, was closely related to the Vietnamese strains. Among the Vietnamese strains, DB2011, LaLc_2011, LamDB_2013, and HG_2015 belonged to the same genetic branch, while TuanDB_2011 and QuyetLC_2011 belonged to independent branches (Figs 3B and 4A). TuanDB_2011 and QuyetLC_2011 clearly possessed additional SNP patterns compared with the other four Vietnamese strains (Fig 3B). We identified seven SNPs specific for Vietnamese strains from different provinces (S2 Table).

The phylogeny from the core genome SNPs by BacWGSTdb revealed that the Vietnamese strains were positioned close to each other within the TEA polytomy for A.Br.011/009 and A.Br.008/011 canSNP lineages, and that Carbosap was closely related to them, as revealed by Parsnp analysis (Fig 3).

Among Vietnamese strains, strains isolated in the same year in 2011 (DB2011, LaLc_2011, TuanDB_2011, and QuyetLC_2011) showed genetic diversity as assessed by the difference in the number of SNPs, as shown in Fig 4B. TuanDB_2011 and QuyetLC_2011 possessed 685 and 688 SNP differences from LaLC_2011, respectively. DB_2011 and HG_2015 had one SNP difference from LaLC_2011 and LamDB_2013 possessed 200 SNP differences from LaLC_2011.

## Discussion

To understand the epidemiological and genetic background of anthrax cases occurring in Vietnam from 2011 to 2015, we surveilled and genetically analyzed *Bacillus anthracis* isolated in northern provinces of Vietnam. Northern mountainous areas such as Lai Châu, Dien Bien, and Ha Giang provinces are considered as high-risk areas for this disease (Fig 1A). The predominant form of human anthrax was cutaneous, but we had not investigated the genetics of *B*. *anthracis* strains isolated from patient skin. To understand the genetic background of *B*. *anthracis* isolated from human cutaneous anthrax, whole-genome analysis of six strains isolated from Lai Châu, Dien Bien, and Ha Giang provinces (Table 1) was performed by a reference genome-based single-nucleotide polymorphism (core genome SNP) strategy, as well as a gene-by-gene genomic analysis (wgMLST) encompassing not only core genes/loci but also accessory genes/loci using Parsnp and BacWGSTdb. This was the first whole-genome analysis of Vietnamese *B*. *anthracis* strains and revealed that they were all classified into the A.Br.011/009 canSNP lineage; no strains from Asia have previously been reported to belong to this lineage. The Carbosap clade that Sahl et al. reported [9] was the nearest genetic clade, which consisted of strains isolated in Italy, France, and Argentina. Seven SNPs specific to Vietnamese *B*. *anthracis* were found using the Parsnp program (S2 Table); however, Vietnamese *B*. *anthracis* strains isolated in the same year, namely, 2001, were rather heterogeneous compared with those isolated in 2013 or 2015 (Figs 3 and 4A). In addition, the strains from the same province showed heterogeneity, contrary to expectations. For example, strains in Dien Bien province, DB_2011, TuanDB_2011, and LamDB_2013, were classified into different branches by core genome SNP analysis (Figs 3B and 4A) and the numbers of SNP differences by wgMLST analysis (Fig 4B). Moreover, strains in Lai Châu province, LaLC_2011 and QuyetLC_2011, belonged to different branches with more than 600 SNP differences between them.

The numbers of SNP differences between strains (Fig 4B) suggested that genetically diverse strains may have expanded in several northern provinces in Vietnam around 2011, however, genetically clonal strains like LamDB_2013 and HG_2015 have spread beyond these provinces since then.

In the present study, whole-genome analysis with a rather small number of strains identified distinct relationships between genetic features and the geographical distribution of strains. Although there was a limited number of strains for genetic analysis, it was interesting to find that strains isolated in northern Vietnam belonged to only one canSNP lineage, with high homogeneity. SNPs specific for strains from different provinces as determined in the present study could be useful for further analysis of isolates. We believe that genomic analysis with detailed epidemiological information and continuous surveillance and sampling of humans, animals, and livestock products could shed light on the infectious source or route into the endemic area in northern Vietnam.

## Supporting information

S1 Table The reference strains of Bacillus anthracis used in the present study belonged to A.Br.011/009 and A.Br.008/011 canSNP lineages. (XLSX)

S2 Table Seven SNPs specific for Vietnamese strains were identified by Parsnp. (XLSX)

S3 Table The list of 4,976 core genome SNPs extracted by Parsnp. (XLSX)
